# Elevated mortality and upregulated SARS-CoV-2-associated pathways in innate and adaptive immune cells from individuals with Down syndrome

**DOI:** 10.1371/journal.pone.0338519

**Published:** 2026-01-05

**Authors:** Daniella Balduino Victorino, Jackeline Moraes Malheiros, Felipe Ten-Caten, Victor Hugo Cardoso Betta, Felipe Farinha Saad Barbosa, Khader Ghneim, Fernanda Caroline Coirada, Fernanda Romano Bruno, Giuliana Xavier de Medeiros, Cassia Silveira, Christina Gavegnano, Robert Balderas, Ana Claudia Brandao, Edward Yang, Helder Nakaya, Gabriela A. Wagner, Luciene Covolan, Susan Pereira Ribeiro

**Affiliations:** 1 Sorbonne Université, Institut du Cerveau - Paris Brain Institute - ICM, Inserm, CNRS, AP-HP, Hôpital de la Pitié Salpêtrière, Paris, France; 2 Department of Physiology, Universidade Federal de Sao Paulo, Sao Paulo, Brazil; 3 Pathology Advanced Translational Research Unit (PATRU), Department of Pathology and Laboratory Medicine, School of Medicine, Emory University, Atlanta, Georgia, United States of America; 4 Department of Infectious and Parasitic Diseases, School of Medicine, University of São Paulo, São Paulo, Brazil; 5 Atlanta VÁ Medical Center, Atlanta, Georgia, United States of America; 6 Emory University Department of Pathology, Atlanta, Georgia, United States of America; 7 Emory University Department of Pharmacology and Chemical Biology, Atlanta, Georgia, United States of America; 8 Emory University Center for Human Health, Atlanta, Georgia, United States of America; 9 Harvard University School of Medicine, Boston, Massachusetts, United States of America; 10 BD Biosciences, San Jose, California, United States of America; 11 Hospital Israelita Albert Einstein, São Paulo, Brazil; 12 Center for Research, Education and Innovation (CEPI), Instituto Jo Clemente, Sao Paulo, Brazil; 13 Departamento de Medicina Preventiva, Universidade Federal de São Paulo, Escola Paulista de Medicina, São Paulo, Brazil; Pennsylvania State University Hershey Medical Center, UNITED STATES OF AMERICA

## Abstract

Trisomy 21 increases the risk of severe outcomes and mortality in hospitalized individuals with Down syndrome (DS) following SARS-CoV-2 infection. Using data from the Brazilian Epidemiological Surveillance Information System Influenza (SIVEP-Gripe), we analyzed 102,767 hospitalized COVID-19 patients (1,115 DS and 101,652 non-DS, NDS). DS patients had a higher prevalence of comorbidities and required ventilatory support, ICU admission, and intubation more frequently than NDS patients (p < 0.001). Mortality was 4.5 times higher in DS patients aged 0–30 years (26.3% vs. 5.9%, p < 0.001) and remained 2.22 times higher after adjusting for comorbidities. DS patients over 30 years also exhibited a 22% increase in mortality (PR 1.22, p < 0.001). Gene expression analysis of pre-pandemic monocytes and T cells from DS individuals revealed upregulated pathways linked to SARS-CoV-2 infection, including interferon signaling and cytokine interactions. This baseline immune dysregulation may contribute to severe COVID-19 outcomes in DS patients. Identifying these altered pathways could inform targeted therapeutic strategies to improve immune homeostasis and clinical outcomes. To the best of our knowledge, this is the first study integrating nationwide clinical outcomes with pre-pandemic immune transcriptomic data to mechanistically explain the heightened COVID-19 severity in individuals with DS.

## Introduction

Down syndrome (DS) is the most common chromosomal disorder worldwide, occurring in nearly 1:700 live births [1]. Increases in childhood survival rates due to better health care, and rising rates of advanced maternal age at conception have contributed to an increase from 49,923 in 1950–206,366 in 2010 [2] cases of DS in the United States. DS incidence is even higher in developing countries where prenatal screening tests and parental decision to terminate pregnancy are not readily available, allowed or culturally accepted [3].

DS is caused by partial or complete trisomy of human chromosome 21 (Hsa21), predisposing affected individuals to the development of heterogeneous ranges of comorbidities [3]. Multiple Hsa21-encoded genes have defined roles in the modulation of innate and adaptive immunity, including four out of six IFN receptor (IFNR) subunits: the Type I IFNR subunits 1 (IFNAR1) and 2 (IFNAR2), the Type II Interferon Gamma Receptor 2 (IFNGR2), and the interleukin (IL) 10 receptor 2 (IL10R2; also known as IL10RB), which serves as a receptor for both Type III IFNR ligands and cytokines such as IL-10, IL-22, and IL-26 [4]. While several mechanisms play a role in gene expression (i.e., epigenetic modifications [5]), the trisomy of Hsa21 is associated to increased inflammation and this could be one of the causes of the exacerbated inflammatory response to SARS-CoV-2, resulting in more severe COVID-19 cases [6]. Indeed, individuals with DS have higher mortality rates from COVID-19 compared to euploid individuals [7–9].

In this article we confirmed the increased mortality rates in DS subjects upon SARS-CoV-2 infection in a large nationwide Brazilian cohort (Brazilian Epidemiological Surveillance Information System Influenza Database), and reanalyzed publicly available bulk RNAseq data sets from sorted monocytes and T cells [10] from healthy Down syndrome and non-Down syndrome (NDS) individuals generated prior to the COVID-19 pandemics. Our data support previous findings in the literature indicating higher mortality rates post-SARS-CoV-2 in individuals with DS. Of note, our data shows that this worse scenario is across all age groups. Furthermore, cells of the innate and adaptive immune system from healthy, uninfected individuals with DS, reveal heightened baseline inflammation being enriched in pathways that are specifically upregulated following SARS-CoV-2 infection. Understanding the upstream pathways involved in the pathophysiology of DS may help identify targetable mechanisms to improve immune system homeostasis, enhance baseline health status, and ensure an adequate immune response to infections.

## Results

### Trisomy 21 is a risk factor for high mortality and severe outcomes following SARS-CoV-2 infection in hospitalized DS subjects

**Fig 1:**
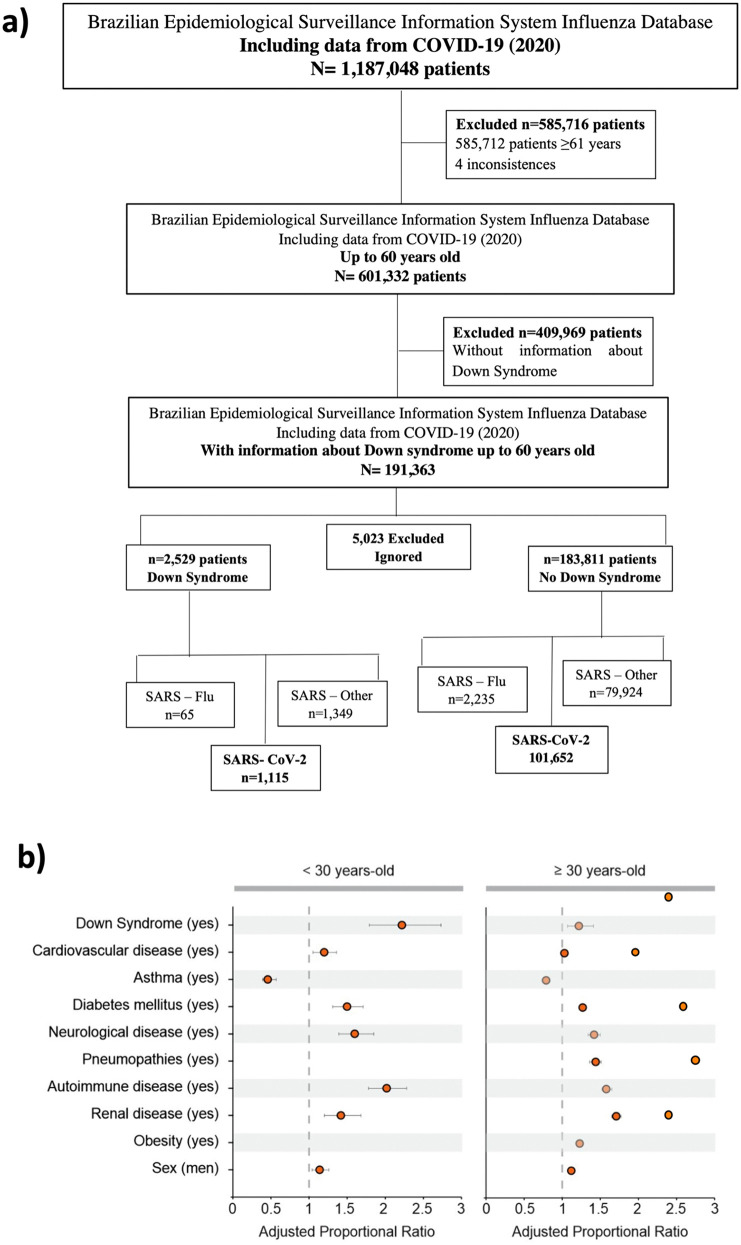
Down Syndrome (DS) subjects present significantly higher ratios of succumbing from COVID-19. **a)** Brazilian Epidemiological Surveillance Information System Influenza (SIVEP-Gripe) data assessment and filtering steps. **b)** Adjusted proportional ratio of DS and other comorbidities for COVID-19 death rates. Age < 30 > years-old (S3 Table).

To compare mortality rates, and clinical outcomes following SARS-CoV-2 infection, hospitalized COVID-19 patients, with and without DS (NDS), were analyzed from a publicly available dataset specifically on SARS (S1 Table) and SARS-CoV-2 cases (S2 and S3 Tables) from the Brazilian Epidemiological Surveillance Information System Influenza (Sistema de Informação de Vigilância Epidemiológica da Gripe-SIVEP-Gripe). Among patients hospitalized between March 1 and November 21/2020, we selected 186,340 patients who were infected with respiratory viral infections (Influenza, SARS, other respiratory viruses and SARS-CoV-2). The selected patients were up to 60 years old to match the maximal age found in DS individuals in this dataset. From those 2,529 were DS and 183.811 were NDS. A total of 5,023 were excluded due to incomplete information. From these individuals, 102,767 were patients with confirmed diagnosis for SARS-CoV-2 infection (1,115 DS and 101,652 NDS) (Fig 1a).

The overall analysis of the sociodemographic characteristics and clinical history of people with DS and NDS up to 60 years hospitalized for overall respiratory viral infections in Brazil at the beginning of the pandemics (2020 – n = 186,340) showed a significant higher prevalence of most of the annotated comorbidities (cardiovascular disease, hematological disease, hepatic disease, neurological disease, pneumopathies, autoimmune disease, renal disease – 0.38 < p < 0.001) in patients with DS versus NDS (S1 Table). Asthma and obesity did not discriminate the groups (p > 0.05). Following hospitalization, the need of ventilatory support, the admission at the intensive care unit (ICU) and endotracheal intubation were more common in patients with DS (p < 0.001 - S1 Table) that were also less likely to recover from respiratory viral infections as compared to those without DS (p < 0.001 –S1 Table).

We next focused specifically on SARS-CoV-2 infected patients (S2 Table). When considering death rates (last column) the mortality was 4.5 times higher in hospitalized COVID-19 patients with DS aged 0–30 years versus NDS at the same age range (p < 0.001–26.3% DS vs 5.9% NDS – highlighted in gray S2 Table). After applying a parsimonious model selecting the variables that best explained the effect of DS on death by a generalized linear model (GML) of Poisson regression with robust variance adjusted by comorbidities and sex by age, hospitalized patients with DS aged 0–30 years still observed a 2.22 times higher mortality from COVID-19 than NDS (highlighted in grey in S3 Table). A 22% increase in mortality was also found among hospitalized COVID-19 patients with DS aged over 30 years when compared to NDS (PR 1.22 – highlighted in grey in S3 Table) (Fig 1b). Our findings corroborate previous evidence of increased susceptibility of people with DS to SARS-CoV-2 clinical complications, including ICU admission, endotracheal intubation, and death [7,8]. However, no conclusion can be drawn about susceptibility to infection.

### Pre-pandemic gene signatures of monocytes from individuals with Down syndrome exhibit upregulated genes associated with SARS-CoV-2 infection

**Fig 2:**
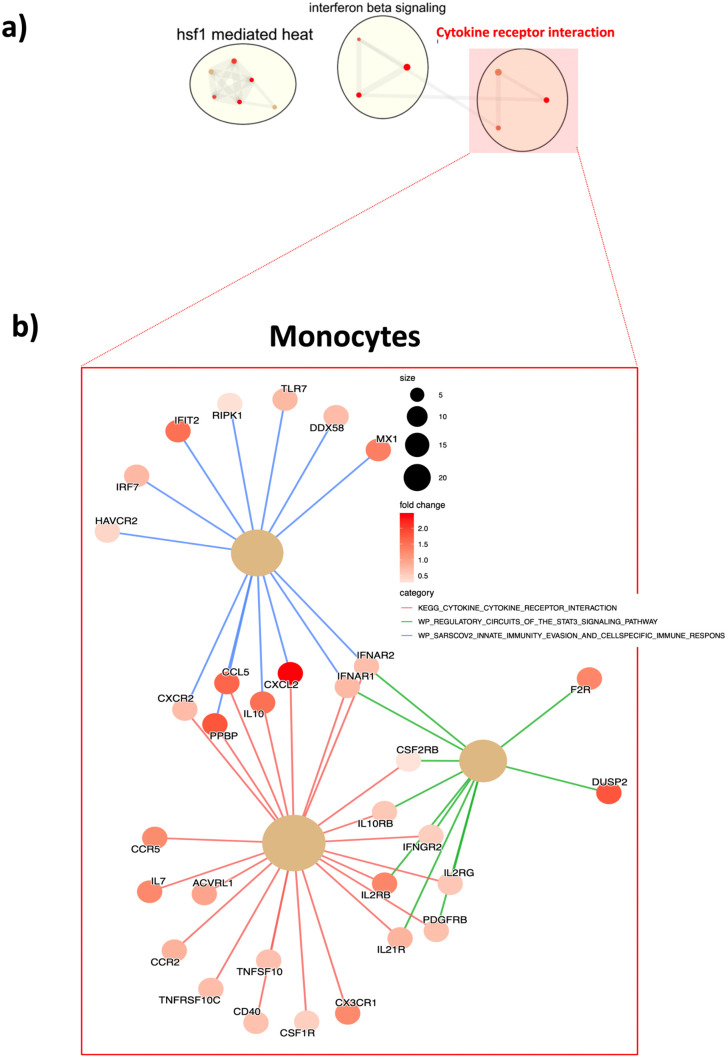
Monocytes from healthy DS individuals present upregulation of pathways associated with SARS infection. **a)** Publicly available data sets [4] from RNAseq on sorted monocytes from DS and NDS subjects were re-analyzed for the modulation of immune pathways. **b)** LEGs (leading edge genes) from pathways associated to SARS-CoV-2 infection are highlighted. Briefly, differential expressed genes (DEGs) were identified using EdgeR package and following default parameters (FDR < 0.1). Enrichr was used to identify upregulated pathways, including pathways identified in NDS upon SARS-Co2 infection. LEGs from SARS-CoV-2 associated pathways (red squares) are plotted (bottom). The size of the letters (top plot) reflects the number of single pathways incorporated in the major pathway cluster. Grey edges (top plot) reflect the association with neighboring pathways. Colored edges (bottom plot) reflect association with the genes and its respective major pathway indicated in the plot legend.

We next wanted to evaluate the baseline status of the innate immune cells from DS as compared to NDS individuals. This knowledge could help us to understand the upstream signals related to the exacerbated inflammatory responses observed in individuals with DS post-infection. To further explore alterations in pathways associated with the innate immunophenotype in DS, we used publicly available datasets on monocytes isolated from healthy subjects with and without DS [10]. Differentially expressed genes (DEGs, p < 0.05) from monocytes from DS vs NDS were identified. We found 730/1292 (57%) genes significantly upregulated in monocytes from DS subjects when compared to those without DS. By applying Enrich R [11] using the significantly upregulated genes in monocytes from DS participants we identified pathways associated with “Hsf1 mediated heat”, “Interferon beta signaling” and “Cytokine receptor interaction”. Interestingly the pathway “Cytokine receptor interaction” included a pathway associated with SARS-CoV-2 named “SARS-CoV-2 innate immunity evasion and cell specific immune responses” (Fig 2a). The leading-edge genes (LEGs) of this pathway includes antiviral genes such as *MX1, DDX58, TRL7, IFIT2, IRF7, IFNAR2, IFNAR1*, among others, all upregulated in DS participants at basal level (Fig 2b and S4 Table). This baseline activation of the innate immune system, specifically in monocytes, in DS subjects may contribute to their higher risk of severe outcomes and increased mortality upon SARS-CoV-2 infection compared to euploidy individuals and were also reported by De Toma and Dierssen, 2021 [12].

### Pre-pandemic gene signatures of T cells from individuals with Down syndrome exhibit upregulated genes associated with SARS-CoV-2 infection

**Fig 3:**
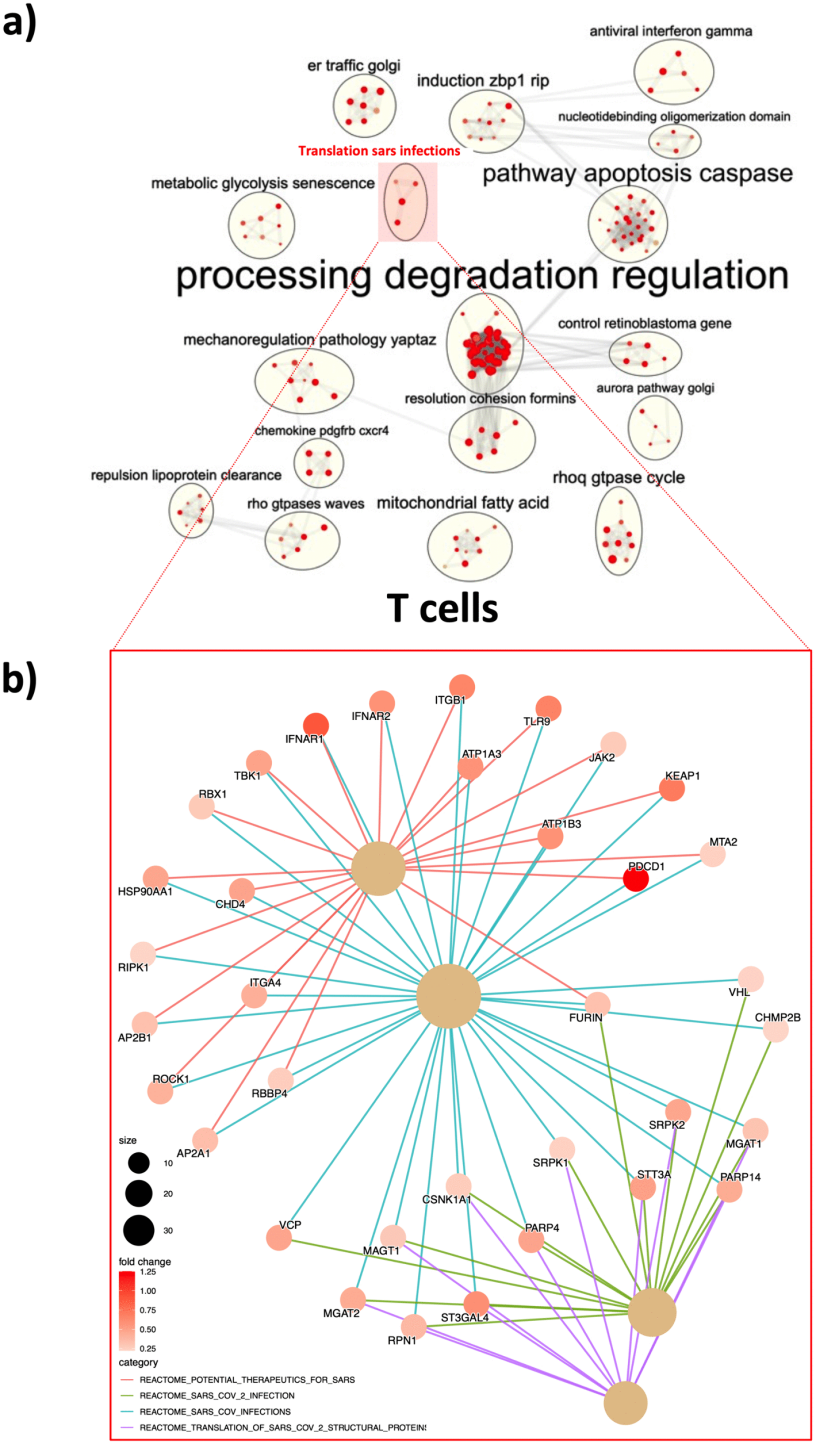
T from healthy DS individuals present upregulation of pathways associated with SARS infection. **a)** Publicly available data sets [4] from RNAseq on sorted T cells from DS and NDS subjects were re-analyzed for the modulation of immune pathways. **b)** LEGs (leading edge genes) from pathways associated to SARS-CoV-2 infection are highlighted. Briefly, differential expressed genes (DEGs) were identified using EdgeR package and following default parameters (FDR < 0.1). Enrichr was used to identify upregulated pathways, including pathways identified in NDS upon SARS-Co2 infection. LEGs from SARS-CoV-2 associated pathways (red squares) are plotted (bottom). The size of the letters (top plot) reflects the number of single pathways incorporated in the major pathway cluster. Grey edges (top plot) reflect the association with neighboring pathways. Colored edges (bottom plot) reflect association with the genes and its respective major pathway indicated in the plot legend.

A strong specific T cell response is crucial for fighting infections and to generate a memory pool able to mountain a recall response in case of re-exposure. Thus, we next evaluated the T cell gene signatures pre-pandemic in individuals with DS as compared to NDS. Corroborating the hyperinflammation profile of the adaptive cells in DS, inspection of publicly available datasets [10], showed that 1714/3184 (54%) genes were upregulated in T cells from healthy DS subjects as compared to those without DS. Following the same strategy as for monocytes, we found that “metabolic glycolysis senescence”, “pathway apoptosis caspase”, processing degradation regulation”, antiviral interferon-gamma”, “translation SARS infections”, among many other pathways (Fig 3a and S5 Table) associated with inflammatory responses against pathogens and protein translation upregulated in T cells from individuals with DS. Of note, the pathway “translation SARS infections” includes several SARS and SARS-CoV-2 pathways extracted from the Reactome data base such as “potential therapeutics for SARS”, “SARS-CoV-2 infection”, SARS-CoV infections” and Translation of SARS-CoV-2 structural protein”. The LEGs of these pathways *are IFNRA1, IFNRA2, JAK2, TBK1, FURIN, CSNK1A1,* among others (Fig 3b). These genes are associated to inflammation and adaptive responses against infections, and negative regulation of canonical Wnt signaling pathway, and they are all enriched in T cells from DS participants as compared to NDS.

## Discussion

Altogether, our data corroborates the heightened mortality score for individuals with DS upon SARS-CoV-2 infection using the *Brazilian Epidemiological Surveillance.* Additionally, it also highlights the higher inflammatory profile in innate (monocytes) and adaptive cells (T cells) from healthy DS participants at pre-pandemic, which are similar to profiles observed upon SARS-CoV-2 infection in NDS participants.

The innate immune system is the first to sense foreign perturbation critical for host defense against pathogens. The PRR (Pattern Recognition Receptors – i.e., TLR, NLR, CLRs) trigger intracellular signaling cascades necessary for transcriptional expression of proinflammatory cytokines and type I interferons (IFN), which are essential for activation of innate cells and subsequent modulation of the adaptive immune responses [13]. IFN-mediated signaling pathways are fundamental for a wide range of immune activities, including host defense against bacterial and viral infections, modulation of inflammatory responses, tissue homeostasis, and tumor immunosurveillance [13]. Sustained activation of these pathways, known as interferonopathies [14], contributes to pathogenesis of many autoinflammatory and autoimmune disorders [13], such as thyroid disease, celiac disease, and type 1 diabetes mellitus (T1DM), common comorbidities in people with DS [3]. While not 100% of DS individuals suffer from interferonopathies, several publications highlight the hyperactivation of the IFN pathway in Down syndrome (DS) participants [10,15–17]. In fact, both immune and non-immune cells obtained from DS subjects were found to be more sensitive to IFN stimulation than those obtained from subjects without DS [10,18,19]. IFN stimulation leads to the activation of Jak/STAT pathways, critical for the upregulation of the downstream inflammatory signals [20]. Indeed, proinflammatory cytokines and chemokines downstream of IFN-induced signaling, such as IL-6, IL-22, MCP-1 (CCL2), TNF-α, and VEGF-A, are upregulated in plasma from individuals with DS, consistent with a hyperactivated IFN paradigm [21,22]. Notably, overexpression of several of these factors has been observed in subjects with autoimmune and autoinflammatory diseases [23,24], and are linked to poor prognosis in COVID-19 [25]. Single-cell transcriptomics of circulating innate immune cells reveal an early antiviral IFN response in all patients with COVID-19 [26]. However, a robust persistent IFN-I response to SARS-CoV-2 may exacerbate inflammation and lead to severe COVID-19 [6,27]. Here we confirm the heightened inflammatory profiles in monocytes from healthy individuals with DS, pre-pandemic, which are similar to the signatures found during SARS-CoV-2 infection.

Similar to innate immunity, the adaptive arm of the immune system in healthy individuals with DS has been shown to be altered. Additional to the overexpression of Hsa21 genes that can lead to hyperinflammation, T and B lymphocytes may receive impaired innate priming, further misguiding the generation of proper adaptive immune responses [28]. A decrease in either frequency or total number of T cells has been observed in the DS population [29–32], with reduced CD4^+^ and normal-to-high CD8^+^ T-cell frequencies (i.e., decreased CD4/CD8 ratio) [18,19,29]. Patients with severe COVID-19 also show lymphocytopenia, which mostly affects the CD4^+^ T-cell subset and correlates with stronger cytokine storm [33–35]. Consistently, increased levels of T-cell-related cytokines (IL-2, MIP-1α, MIP-3α, IL-10, Eotaxin, IL-8, IL-17A–D, and IL-22) have been found in plasma of individuals with DS [18]; among them IL-10 and IL-8 are upregulated in severe COVID-19 [36]. The expression of markers associated with T-cell activation, differentiation, proliferation, and senescence are altered in persons with DS [37], which is similar to what has been observed in severe COVID-19, wherein increased expression of exhaustion-related modules (PD-1, CTLA-4, Tim-3, and TIGIT in CD8^+^ T cells) has been found [38–40], and also reported to be downstream of IFN signaling [41]. These findings suggest that severe COVID-19 damages the function of CD4^+^ T cells and promotes excessive CD8^+^ T-cell activation and exhaustion, which may impair specific T-cell responses against SARS-CoV-2, mimicking the dysregulated profiles of immune responses observed in DS subjects. In addition, regulatory T cells (Treg), known to control the magnitude of the host immune responses, has been reported to be impaired in DS individuals, supporting the immune dysfunction leading to autoimmune and inflammatory diseases [42]. Increased proportions of dysfunctional Tregs, combined with the expression of proinflammatory mediators, were shown to distinguish severe COVID-19 [43] to mild to moderate cases. Here we confirm the heightened inflammatory profiles in T cells from healthy individuals with DS, pre-pandemic, which are similar to the signatures found during SARS-CoV-2 infection.

These data underscore the collective dysregulation that originates from the chromosome 21 trisomy, rendering individuals with DS more likely to suffer from severe COVID-19 disease and related mortality [44]. Baricitinib is a Jak 1/2 selective inhibitor originally approved for rheumatoid arthritis [45]; baricitinib and the first-generation Jak ½ inhibitor, ruxolitinib, were studied extensively in the viral infection setting, including in people living with HIV [46,47], demonstrating reversal of immune dysfunction that drives disease pathogenesis/severity and related mechanistic effects mitigating disease progression [46,47]. Baricitinib was repurposed for COVID-19, receiving EUA for hospitalized COVID-19 patients requiring supplemental oxygen (11/2020). Recently, the World Health Organization (WHO) conferred a “strong recommendation” for worldwide use of baricitinib for COVID-19 in hospitalized patients, and the RECOVERY Trial (>8,000 patients) demonstrated that baricitinib confers a significant mortality benefit for critically-ill COVID-19 patients, when added to any standard of care [48]. This mortality benefit is linked to reduction in the cytokine storm, reversal of immune dysfunction, and restoration of immune homeostasis [48–50]. This paradigm is magnified in DS, where the immune deregulatory cascade is altered at baseline due to trisomy 21. Collectively, baricitinib may represent a treatment for DS patients with COVID-19. However, considering the recent publication by Malle et al., [51] and nicely discussed by Notarangelo and Bosticardo [52], the negative feedback loop of IFN-signaling needs to be taken in consideration to avoid further decrease of the antiviral cascade in these individuals upon baricitinib intake. Importantly, other management approaches should take place such as priority for prophylactic immunization and/or therapeutic administration of anti- SARS-CoV-2 monoclonal antibodies, with the latter being cautiously used due to the resistance of the new circulating variants to the prior developed neutralizing antibodies [53–55].

We showed that several immune perturbations associated with severe COVID-19 are similar to multiple DS-related immunological features. The chronic state of inflammation, in concert with upregulation of genes reported to be involved in SARS-CoV-2 pathogenesis, may contribute to exacerbated and uncontrolled immune response in individuals with DS upon SARS-CoV-2 infection, thus conferring predisposition to severe COVID-19. However, several other mechanisms could play a role in this worse scenario post-SARS-CoV-2 infection, including the presence of auto-IFN antibodies [56,57]. Importantly, Malle et al. [7], have recently reported that increased susceptibility to severe infections could be a result of dysregulated IFN-I responses with increased initial signaling translating into a state a refractoriness, based on the expression of the protein USP18, that makes the immune systems of DS individuals less capable of controlling viral infections. Nevertheless, this data needs to be further supported by *in vivo/ex vivo* data during active infection in blood cells and possibly upper respiratory tract cells from DS participants, as discussed by Notarangelo and Bosticardo [52]. Additionally, while still not reported in the literature, DS individuals could be more predisposed to long-COVID or post-acute COVID-19 syndrome (PACS) [58,59]. Although mechanisms of long-COVID development are incompletely understood, increased hospitalization rate, comorbidities and number of symptoms presented during first week of illness were associated with the risk of long-COVID development [60,61], underscoring the likelihood of PACS or long-COVID in DS subjects. However, up to now few cases of PASC were reported in the literature [51] in DS individuals and led to a severe form of with multisystem inflammatory syndrome in children (MIS-C). Improved understanding of how trisomy 21 confers poor COVID-19 outcomes is paramount towards mitigation of increased mortality rates among people with DS, and for informed, paired public-health measures. Herein, we describe and validate in a large Brazilian cohort, that DS subjects in different age ranges experience more severe COVID-19. These subjects, in healthy state, present a pro-inflammatory milieu akin to observations upon SARS-CoV-2 infection, which may predispose them to a higher mortality risk. Importantly, we hypothesize and discuss that gut microbiome–metabolome interactions, their impact on epigenetics and trained immunity, and subsequent shaping of adaptive immune responses, are altered in these subjects. Early interventions upon SARS-CoV-2 infection are critical to prevent mortality and possibly to avoid long-COVID-19 symptoms in these subjects. Intervention with baricitinib warrants investigation to better understand its potential role in treatment of people living with DS in the viral infection setting.

## Limitations

The data sets from the “Brazilian Epidemiological Surveillance Information System Influenza” herein used was created during the COVID-19 Pandemic Public Emergency in 2020. This was an initiative of the Brazilian Ministry of Health, and it involves cases from the entire health network in Brazil. Nevertheless, the data collection was susceptible to errors and sub notifications as most of the secondary data, such as comorbidities, was inferred by self-reporting and not actual diagnostic tests or clinical evaluation; and several relevant fields are missing (i.e., hypothyroidism) and/or sub notified by lack of knowledge which might limit the correction for confounding factors.

## Future directions

Our findings highlight the disproportionate risk of severe COVID-19 outcomes and mortality among individuals with Down syndrome, underpinned by baseline immune dysregulation detectable in both innate and adaptive compartments. Future studies should expand longitudinal cohorts to assess whether these transcriptomic signatures persist across different age groups, viral variants, and vaccination statuses. Functional assays are warranted to dissect how interferon signaling and cytokine receptor pathways contribute to hyperinflammation and impaired viral clearance in DS. Moreover, integrating single-cell multi-omics and systems immunology approaches could help unravel cell type–specific vulnerabilities. Importantly, targeted therapeutic strategies aiming to restore immune homeostasis—such as modulation of interferon responses or metabolic pathways—should be investigated in preclinical and clinical settings to reduce the burden of infectious diseases in this high-risk population.

## Methods

### Epidemiological study

#### Design.

To evaluate if DS individuals were at higher risk of adverse outcomes post-SARS-CoV-2 infection in Brazil, we analyzed SARS-CoV-2 reported on the Brazilian Epidemiological Surveillance Information System Influenza (SIVEP-Gripe) from Ministry of Health, focusing on cases reported in the beginning of the pandemics (2020). Cross-sectional cases with secondary data entry of SARS notification records of hospitalized cases were filtered. The COVID-19 surveillance in Brazil was incorporated into the influenza and other respiratory virus surveillance network. SARS cases are defined by individuals who met the following criteria: (i) individual hospitalized with self-reported or measured fever; (ii) cough or sore throat; (iii) dyspnea or O2 saturation <95% or respiratory distress; and (iv) need for hospitalization, or evolution to death, having presented the aforementioned symptoms, regardless of hospitalization (MS, 2020 - https://s3.sa-east-1.amazonaws.com/ckan.saude.gov.br/SRAG/2020/INFLUD20-26-06-2025.csv).

#### Inclusion criteria.

Cases under 60 years old with notification of Down syndrome vs NDS. Individuals over 61 years old and those without DS notification were excluded (Fig 1a). The prevalence of COVID-19 in DS patients was estimated from the variable: Final classification of the case categorized into: SARS – Influenza; SARS – Other viruses and SARS-CoV-2. Considered outcomes were i) recovered, ii) intensive care unit or iii) death. Covariates such as sex, age (0–30 and ≥30), race and Brazilian macro-region were analyzed, in addition to the comorbidities self-reported in the notification records (cardiovascular disease, hematological disease, hepatic disease, asthma, diabetes mellitus, neurological disease, pneumopathies, autoimmune disease, renal disease and obesity) were taken in consideration for the evaluation of the outcomes.

#### Statistical analyses.

The relationship between sociodemographic and clinical history by outcomes (recovered, intensive care unit and death) of people with DS and NDS was defined by Pearson’s chi-square test. A parsimonious model was applied, which selected the variables that best explained the effect of DS on death by a generalized linear model (GML) of Poisson regression with robust variance adjusted by comorbidities and sex by age. Strength of association was evaluated by proportional ratio (PR), with its respective 95% confidence intervals (95%CI) (p < 0.05). Statistical data processing was performed in Stata 17®.

### RNA-seq analysis

Publicly available data sets [10] from RNAseq on sorted monocytes and T cells from DS and NDS subjects were re-analyzed for the modulation of immune pathways. Differentially expressed genes (DEGs) were identified using EdgeR package and following default parameters (FDR < 0.1). Pathway over-representation analysis was performed with Enricher function (clusterProfiler 4.6.0) and MSigDB C2 curated gene sets (v7.5.1). Significant pathways (Benjamini-Hochberg BH < 0.05) were clustered, based on the Jaccard similarity coefficient, and clusters were automatically annotated with AutoAnnotate app (v1.3.5) on Cytoscape (c 3.9.1). Figs 2 and 3 are the over-represented pathways that include ≥3 single pathways.

Genes from clusters of interest – selected were the ones that include SARS or SARS-CoV-2 induced pathways and its functional association was further explored by using http://bioconductor.org/packages/release/bioc/html/enrichplot.html.

## Supporting information

S1 Table Sociodemographic characteristics and clinical history of people with Down syndrome (DS) and without DS (NDS) up to 60 years hospitalized for SARS infection in Brazil – 2020 (n = 186,340). (DOCX)

S2 Table Sociodemographic characteristics and clinical history by COVID-19 outcomes of people with Down syndrome (DS) and without DS (NDS) up to 60 years hospitalized for SARS-CoV-2 in Brazil – 2020 (n = 102,767). (DOCX)

S3 Table Generalized linear adjustment model of Poisson regression with robust variance including selected variables that best explain the effect of Down syndrome on death and ICU admission followed by death due to SARS-CoV-2 infection. (DOCX)

S4 Table Monocytes up-regulated pathways and clustering. (XLSX)

S5 Table T cells up-regulated pathways and clustering. (XLSX)
