# Supplementary material for: Elevated mortality and upregulated SARS-CoV-2-associated pathways in innate and adaptive immune cells from individuals with Down syndrome
Source: PLoS One. 2026 Jan 5;21(1):e0338519. doi: 10.1371/journal.pone.0338519 (PMC12768363; doi:10.1371/journal.pone.0338519)
Supplement: S1 Table — (DOCX) [file pone.0338519.s001.docx]

**Table S1.** Sociodemographic characteristics and clinical history of people with Down syndrome (DS) and without DS (NDS) up to 60 years hospitalized for SARS infection in Brazil – 2020 (n=186,340).

|  | **NDS%(n)** | **DS%(n)** | **p-value*** |
| --- | --- | --- | --- |
|  | 98.6 (183,811) | 1.4 (2,529) |  |
| **Sex** |  |  |  |
| Women | 46.1(84,635) | 46.7(1,182) | 0.487 |
| Men | 53.9(99,107) | 53.2(1,346) |  |
| **Age (years)** |  |  | **<0.001** |
| 0-30 | 18.5(34,042) | 46.9(1,185) |  |
| ≥30 | 81.5(149,769) | 53.1(1,344) |  |
| **Ethnicity/Color** |  |  | **<0.001** |
| White | 40.8 (73,070) | 47.9 (1,164) |  |
| African-Brazilians | 44.1(78,995) | 36.2(881) |  |
| Multi-ethnic group | 0.9(1,681) | 0.8(19) |  |
| Indigenous Peoples | 0.3(469) | 0.5 (13) |  |
| Not declared | 14.0(25,033) | 14.6(354) |  |
| **Brazilian region** |  |  |  |
| South | 17.0(31,211) | 17.0(430) | **0.001** |
| Southeast | 50.5(92,864) | 50.2(1,269) |  |
| Midwest | 10.2(18,705) | 8.0(203) |  |
| North | 6.1(11,251) | 7.1(179) |  |
| Northeast | 16.2(29,780) | 17.7(448) |  |
| **Cardiovascular disease** |  |  | **<0.001** |
| No | 63.7(116,535) | 59.9(1,213) |  |
| Yes | 36.3(66,511) | 40.1(813) |  |
| **Hematological disease** |  |  | **<0.001** |
| No | 98.1(179,175) | 92.4(1,722) |  |
| Yes | 1.9(3,443) | 7.6(142) |  |
| **Hepatic disease** |  |  | **<0.001** |
| No | 97.9(178,245) | 93.5(1,743) |  |
| Yes | 2.1(3,383) | 6.5(122) |  |
| **Asthma** |  |  | 0.706 |
| No | 89.4(162,786) | 89.6(1,688) |  |
| Yes | 10.6(19,352) | 10.4(195) |  |
| **Diabetes mellitus** |  |  | **<0.001** |
| No | 71.7(130,979) | 79.9(1,533) |  |
| Yes | 28.3(51,789) | 20.1(386) |  |
| **Neurological disease** |  |  | **<0.001** |
| No | 94.4(172,093) | 86.6(1,642) |  |
| Yes | 5.6(10,197) | 13.4(255) |  |
| **Pneumopathies** |  |  | **<0.001** |
| No | 94.3(171,885) | 88.9(1,664) |  |
| Yes | 5.7(10,414) | 11.1(208) |  |
| **Autoimmune disease** |  |  | **0.002** |
| No | 92.1(167,312) | 90.1(1,695) |  |
| Yes | 7.9(14,436) | 9.9(186) |  |
| **Renal disease** |  |  | **0.038** |
| No | 94.0(170,450) | 92.9(1,730) |  |
| Yes | 6.0(10,867) | 7.1(133) |  |
| **Obesity** |  |  | 0.163 |
| No | 87.6(156,457) | 86.5(1,611) |  |
| Yes | 12.4(22,256) | 13.5(252) |  |
| **SARS** |  |  | **<0.001** |
| Influenza | 1.3(2,235) | 2.8(65) |  |
| Other virus | 40.4(70,529) | 49.9(1,175) |  |
| SARS-CoV-2 | 58.3(101,652) | 47.3(1,115) |  |
| **Intensive Care Unit** |  |  | **<0.001** |
| No | 66.9(113,412) | 57.9(1,328) |  |
| Yes | 33.1(56,112) | 42.1(964) |  |
| **Ventilatory support** |  |  | **<0.001** |
| No | 32.9(55,333) | 27.5(622) |  |
| Non-invasive ventilation | 49.9(83,922) | 48.1(1,086) |  |
| Endotracheal Intubation | 17.2(28,978) | 24.4(550) |  |
| **Outcome** |  |  | **<0.001** |
| Recovered | 78.4(125,973) | 71.5(1,542) |  |
| Death | 21.6(34,813) | 28.5(615) |  |

**Source:** SRAG 2020 - Brazilian Epidemiological Surveillance Information System Influenza Database - Including data from COVID-19 (2020). Available in: https://s3.sa-east-1.amazonaws.com/ckan.saude.gov.br/SRAG/2020/INFLUD20-26-06-2025.csv. *p-value: Chi-square for categorical variables.
