# Supplementary material for: Elevated mortality and upregulated SARS-CoV-2-associated pathways in innate and adaptive immune cells from individuals with Down syndrome
Source: PLoS One. 2026 Jan 5;21(1):e0338519. doi: 10.1371/journal.pone.0338519 (PMC12768363; doi:10.1371/journal.pone.0338519)
Supplement: S2 Table — (DOCX) [file pone.0338519.s002.docx]

**sTable S2.** Sociodemographic characteristics and clinical history by COVID-19 outcomes of people with Down syndrome (DS) and without DS (NDS) up to 60 years hospitalized for SARS-CoV-2 in Brazil – 2020 (n=102,767).

|  | **Recovered** | | | **Intensive Care Unit** | | | **Death** | | |
| --- | --- | --- | --- | --- | --- | --- | --- | --- | --- |
|  | **NDS%(n)** | **DS%(n)** | **p-value** | **NDS%(n)** | **DS%(n)** | **p-value** | **NDS%(n)** | **DS%(n)** | **p-value** |
| **Sex** |  |  | 0.087 |  |  | 0.220 |  |  | **0.001** |
| Women | 46.3(22,775) | 45.9(184) |  | 41.9(7,318) | 46.3(88) |  | 41.1(8,619) | 49.7(168) |  |
| Men | 53.7(26,378) | 54.1(217) |  | 58.1(10,146) | 53.7(102) |  | 58.9(12,340) | 50.3(170) |  |
| **Age (years)** |  |  | **<0.001** |  |  | <0.001 |  |  | **<0.001** |
| 0-30 | 9.8(4,813) | 28.7(115) |  | 9.2(1,711) | 36.3(69) |  | ***5.9(1,237)*** | ***26.3(89)*** |  |
| ≥30 | 90.2(44,344) | 71.3(286) |  | 90.2(15,755) | 63.7(121) |  | 94.1(19,726) | 73.7(249) |  |
| **Ethnicity/Color** |  |  | 0.099 |  |  | 0.071 |  |  | **0.043** |
| White | 44.0(21,149) | 50.8(196) |  | 45.4(7,768) | 51.6(97) |  | 38.2(7,825) | 45.2(146) |  |
| African-Brazilians | 40.3(19,355) | 34.2(132) |  | 35.9(6,143) | 27.1(51) |  | 48.3(9,901) | 41.8(135) |  |
| Multi-ethnic group | 1.0(498) | 0.8(3) |  | 0.8(136) | 0 |  | 1.0(194) | 0.9(3) |  |
| Indigenous Peoples | 0.3(118) | 0.3(1) |  | 0.2(26) | 0 |  | 0.4(75) | 0.9(3) |  |
| Not declared | 14.4(6,923) | 14.0(54) |  | 17.8(3,039) | 21.3(40) |  | 12.2(2,488) | 11.2(36) |  |
| **Brazilian region** |  |  | 0.134 |  |  | 0.610 |  |  | 0.390 |
| South | 18.6(9,123) | 18.2(73) |  | 18.2(3,181) | 18.4(35) |  | 15.0(3,133) | 13,9(47) |  |
| Southeast | 49.4(24,287) | 52.4(210) |  | 52.9(9,232) | 48.9(93) |  | 44.6(9,358) | 45.6(154) |  |
| Midwest | 11.2(5,485) | 7.2(29) |  | 11.5(2,008) | 11.0(21) |  | 10.6(2,224) | 7.7(26) |  |
| North | 7.3(3,576) | 8.5(34) |  | 3.5(609) | 3.7(7) |  | 9.8(2,049) | 11.2(38) |  |
| North East | 13.6(6,686) | 13.7(55) |  | 13.9(2,436) | 17.9(34) |  | 20.0(4,199) | 21.6(73) |  |
| **Cardiovascular disease** |  |  | 0.058 |  |  | 0.028 |  |  | **0.010** |
| No | 58.6(28,719) | 63.8(211) |  | 56.6(9,861) | 65.4(102) |  | 55.3(11,522) | 63.3(167) |  |
| Yes | 41.4(20,292) | 36.2(120) |  | 43.4(7,554) | 34.6(54) |  | 44.7(9,320) | 36.7(97) |  |
| **Hematological disease** |  |  | **<0.001** |  |  | <0.001 |  |  | **<0.001** |
| No | 98.7(48,272) | 87.4(271) |  | 98.9(17,179) | 88.8(127) |  | 98.0(20,356) | 92.3(229) |  |
| Yes | 1.3(654) | 12.6(39) |  | 1.1(189) | 11.2(16) |  | 2.0(415) | 7.7(19) |  |
| **Hepatic disease** |  |  | **<0.001** |  |  | <0.001 |  |  | **0.005** |
| No | 98.7(48,119) | 89.7(278) |  | 98.6(17,176) | 90.9(130) |  | 96.7(20,041) | 93.4(228) |  |
| Yes | 1.3(656) | 10.3(32) |  | 1.3(229) | 9.1(13) |  | 3.3(680) | 6.6(16) |  |
| **Asthma** |  |  | **0.010** |  |  | 0.201 |  |  | 0.107 |
| No | 92.3(45,042) | 88.4(275) |  | 93.1(16,137) | 90.3(131) |  | 95.3(19,728) | 93.1(228) |  |
| Yes | 7.7(3,742) | 11.6(36) |  | 6.9(1,204) | 9.7(14) |  | 4.7(980) | 6.9(17) |  |
| **Diabetes mellitus** |  |  | 0.201 |  |  | 0.432 |  |  | **<0.001** |
| No | 67.9(33,231) | 71.3(226) |  | 66.3(11,528) | 69.3(104) |  | 59.3(12,341) | 75.5(191) |  |
| Yes | 32.1(15,687) | 27.3(91) |  | 33.7(5,862) | 30.7(46) |  | 40.7(8,484) | 24.5(62) |  |
| **Neurological disease** |  |  | **<0.001** |  |  | <0.001 |  |  | **<0.001** |
| No | 97.1(47.422) | 86.1(267) |  | 96.3(16,689) | 88.0(125) |  | 95.0(19,690) | 85.9(213) |  |
| Yes | 2.9(1,395) | 13.9(43) |  | 3.7(644) | 12.0(17) |  | 5.0(1,028) | 14.1(35) |  |
| **Pneumopathies** |  |  | **<0.001** |  |  | 0.001 |  |  | 0.322 |
| No | 97.0(47,370) | 90.9(280) |  | 96.6(16,759) | 91.7(132) |  | 94.8(19,661) | 93.4(225) |  |
| Yes | 3.0(1,480) | 9.1(28) |  | 3.4(586) | 8.3(12) |  | 5.2(1,081) | 6.6(16) |  |
| **Autoimmune disease** |  |  | **<0.001** |  |  | 0.002 |  |  | 0.591 |
| No | 95.2(46,333) | 89.9(278) |  | 95.7(16,534) | 90.4(132) |  | 90.8(18,756) | 89.8(219) |  |
| Yes | 4.8(2,333) | 10.1(31) |  | 4.3(742) | 9.6(14) |  | 9.2(1,910) | 10.3(25) |  |
| **Renal disease** |  |  | **<0.001** |  |  | 0.616 |  |  | 0.082 |
| No | 96.0(46,678) | 89.0(275) |  | 94.8(16,340) | 95.7(134) |  | 89.6(18,496) | 93.0(227) |  |
| Yes | 4.0(1,956) | 11.0(34) |  | 5.2(902) | 6(4) |  | 10.4(2,141) | 7.0(17) |  |
| **Obesity** |  |  | 0.547 |  |  | 0.341 |  |  | 0.168 |
| No | 85.0(40,671) | 86.2(257) |  | 78.1(13,267) | 81.4(118) |  | 80.5(16,156) | 77.0(194) |  |
| Yes | 15.0(7,181) | 13.8(41) |  | 21.9(3,721) | 18.6(27) |  | 19.6(3,926) | 23.0(58) |  |
| **Ventilatory support** |  |  | 0.421 |  |  | 0.052 |  |  | 0.709 |
| No | 38.7(18,043) | 37.0(138) |  | 16.6(2,776) | 16.0(29) |  | 10.0(2,003) | 8.7(28) |  |
| Non-invasive ventilation | 59.4(27,682) | 60.3(225) |  | 58.6(9,798) | 51.4(93) |  | 33.5(6,682) | 33.3(107) |  |
| Endotracheal Intubation | 1.9(860) | 2.7(10) |  | 24.9(4,156) | 32.6(59) |  | 56.4(11,246) | 58.0(186) |  |

**Source:** SRAG 2020 - Brazilian Epidemiological Surveillance Information System Influenza Database - Including data from COVID-19 (2020). Available in: https://s3.sa-east-1.amazonaws.com/ckan.saude.gov.br/SRAG/2020/INFLUD20-26-06-2025.csv. *p-value: Chi-square for categorical variables.
