# Supplementary material for: Elevated mortality and upregulated SARS-CoV-2-associated pathways in innate and adaptive immune cells from individuals with Down syndrome
Source: PLoS One. 2026 Jan 5;21(1):e0338519. doi: 10.1371/journal.pone.0338519 (PMC12768363; doi:10.1371/journal.pone.0338519)
Supplement: S3 Table — (DOCX) [file pone.0338519.s003.docx]

**Table S3.** Generalized linear adjustment model of Poisson regression with robust variance including selected variables that best explain the effect of Down syndrome on death and ICU admission followed by death due to SARS-CoV-2 infection.

|  | **DEATH** | | | |
| --- | --- | --- | --- | --- |
|  | **0-30** | | **≥30** | |
|  | **UnAdjPR^1^** | **AdjPR^2^** | **UnAdjPR^1^** | **AdjPR^2^** |
| **Down syndrome** |  |  |  |  |
| Yes | 2.12(1.80-2.50) | 2.22(1.79-2.73) | 1.53(1.40-1.68) | 1.22(1.07-1.41) |
| **Cardiovascular disease** |  |  |  |  |
| Yes | 1.33(1.18-1.49) | 1.20(1.05-1.36) | 1.04(1.01-1.06) | 1.03(1.01-1.06) |
| **Hepatic disease** |  |  |  |  |
| Yes | 1.43(1.00-2.03) | - | 1.77(1.68-1.88) | - |
| **Asthma** |  |  |  |  |
| Yes | 0.37(0.31-0.46) | 0.46(0.39-0.57) | 0.76(0.72-0.80) | 0.79(0.74-0.84) |
| **Diabetes mellitus** |  |  |  |  |
| Yes | 1.45(1.28-1.65) | 1.50(1.31-1.71) | 1.26(1.23-1.29) | 1.27(1.24-1.30) |
| **Neurological disease** |  |  |  |  |
| Yes | 1.53(1.33-1.76) | 1.60(1.39-1.85) | 1.48(1.40-1.56) | 1.42(1.34-1.50) |
| **Pneumopathies** |  |  |  |  |
| Yes | 1.07(0.85-1.34) | - | 1.48(1.41-1.55) | 1.44(1.36-1.51) |
| **Autoimmune disease** |  |  |  |  |
| Yes | 2.05(1.85-2.30) | 2.02(1.78-2.28) | 1.63(1.57-1.70) | 1.58(1.52-1.65) |
| **Renal disease** |  |  |  |  |
| Yes | 1.63(1.39-1.91) | 1.42(1.20-1.68) | 1.86(1.80-1.92) | 1.71(1.65-1.77) |
| **Obesity** |  |  |  |  |
| Yes | 0.96(0.85-1.09) | - | 1.17(1.13-1.20) | 1.23(1.19-1.27) |
| **Sex** |  |  |  |  |
| Men | 1.14(1.08-1.20) | 1.14(1.04-1.26) | 1.11(1.08-1.14) | 1.12(1.09-1.15) |

**Source:** SRAG 2020 - Brazilian Epidemiological Surveillance Information System Influenza Database - Including data from COVID-19 (2020). Available in: https://s3.sa-east-1.amazonaws.com/ckan.saude.gov.br/SRAG/2020/INFLUD20-26-06-2025.csv.

1. PR: Proportional Ratio

2. Generalized linear adjustment model of Poisson regression with robust variance adjusted by comorbidities and sex.
